# Hydraulic function of medullary bundles in a tropical root climber

**DOI:** 10.1002/ajb2.70256

**Published:** 2026-09-04

**Authors:** Sandrine Isnard, Damien Longepierre, Taylor Feild

**Affiliations:** ^1^ AMAP, Université Montpellier, IRD, CIRAD, CNRS, INRAE Montpellier France; ^2^ CEFE, Université Montpellier, CNRS, EPHE, IRD Montpellier France; ^3^ Department of Natural Sciences New College of Florida Sarasota FL USA

**Keywords:** hydraulic architecture, leaf trace, medullar conductivity, medullary bundles, Melastomataceae, monocot‐like anatomy, nomadic vine, root climber, stem capacitance

## Abstract

**Premise:**

Medullary bundles, which are vascular bundles in the pith, have evolved multiple times. However, their functional significance remains poorly understood. Here, we investigated how medullary bundles are involved in the vascular architecture and hydraulic function in a tropical root climber, *Adelobotrys scandens* (Melastomataceae) to understand the functional significance of this vascular architecture in a wet‐adapted root climber species as compared to what is known about medullary bundles in the lineages in hydraulically limited arid/saline environments.

**Methods:**

We used light microscopy to examine the organization of the medullary bundles in the stem. We measured the hydraulic conductivity of intact stems and compared it with those of stems that were experimentally reduced to pith tissue. These analyses allowed estimation of hydraulic conductivity through the pith tissue system.

**Results:**

*Adelobotrys adscendens* has a polycyclic eustele, with procambium‐derived medullary bundles of two types: peripheric bundles forming leaf traces and cauline bundles forming sympodia at nodal regions of stems. Although the pith has low intrinsic hydraulic conductivity, pith tissues contributed up to 80% of total stem hydraulic conductivity near the apex, before secondary xylem becomes the dominant hydraulic pathway.

**Conclusions:**

Our experimental study enhances the understanding of atypical vasculature in woody plants. We demonstrated that medullary bundles function in plant water balance, particularly in water transport processes in leaf traces and young shoots, where the pith dominates stem cross‐sectional investment. We discuss how this type of vasculature is involved in the evolution of life forms that develop aerial adventitious roots.

The xylem vascular architecture is an essential aspect of plant hydraulic function, determining how water and nutrients are transported, stored, and redistributed among organs that comprise whole plants (Zimmermann, 1983). In most non‐monocot angiosperms, primary vascular bundles develop in a single ring, with the pith developed toward the center of the stem and a cortex region developed toward the exterior (Esau, 1958). Several angiosperm lineages, however, diverge from this widespread organizational pattern by producing additional primary vascular bundles within the pith. Such atypical vascular bundles are referred to as medullary bundles (Holwill, 1950; Metcalfe and Chalk, 1950; Fahn, 1967).

Medullary bundles have evolved repeatedly across angiosperms, and they exhibit diverse developmental origins (Wilson, 1924; Metcalfe and Chalk, 1950; Mauseth, 1993; Isnard et al., 2012; Cunha Neto et al., 2020, 2024). Their ontogenetic origins range from procambial strands near the shoot apex to extrafascicular strands that differentiate later in development, and perimedullary strands that are initiated along the periphery of the pith in a manner that is often asynchronous with respect to primary growth (Wilson, 1924; Mauseth, 1993; Evert, 2006; Cunha Neto et al., 2024). Furthermore, medullary bundles are structurally diverse. As examples, they can be collateral (having primary xylem internal to primary phloem), amphivasal (primary xylem enclosing primary phloem), arranged in isolated strands, in multiple rings, or as components of more complex polystelic configurations (Wilson, 1924; Dastur, 1925; Joshi, 1934; Beck et al., 1982; Mauseth, 1993; Isnard et al., 2012; Trueba et al., 2015; Cunha Neto et al., 2024). In some taxa (a few members of the Caryophyllales and Piperaceae), medullary bundles develop secondary vascular tissues within the pith (Yang and Chen, 2017; Cunha Neto et al., 2024). Medullary bundles, in some taxa, can be viewed as a type of vascular variant, i.e., having an alternative ontogenetic origin of the vascular tissues (compared to the regular vascular development) (Cunha Neto, 2023). Deviations from typical vascular development are frequent in climbers (Angyalossy et al., 2015; Isnard and Feild, 2015; Cunha Neto, 2023; Kaçamak et al., 2025).

Medullary bundles have been documented across ~40 families of angiosperms (Metcalfe and Chalk, 1950). In some lineages, medullary bundles are rare, occurring in a few isolated species. However, in other lineages, medullary bundles are pervasive. As an example of the latter, medullary bundles are diverse in the Caryophyllales, involving numerous independent origins with varied structural aspects (Cunha Neto et al., 2024). Piperaceae represent another lineage marked by tremendous medullary bundle diversity, where polystelic arrangements and concentric rings of medullary bundles are common (Beck et al., 1982; Isnard et al., 2012; Trueba et al., 2015).

Interestingly, the occurrence of medullary bundles correlates with specific growth habits, including herbs of saline and drought‐prone habitats, succulents (Cactaceae), climbers, and epiphytes (Wilson, 1924; Mauseth, 1993; Isnard et al., 2012; Trueba et al., 2015; Cunha Neto et al., 2020, 2024). These associations suggest a functional linkage between medullary bundles and possibly hydraulic water balance (Wilson, 1924; Esau, 1958; Mauseth, 1993). However, the functional significance of medullary bundles in stem hydraulic properties remains poorly understood.

Additional evidence supports a function of medullary bundles in axial hydraulic flow (Cunha Neto et al., 2024). Medullary bundles appear to be hydraulically capable, based on observations of dye perfusion, of moving water axially during transpiration and calculation of theoretical conductivity in three species (Cunha Neto et al., 2024). These observations suggest that a hydraulic pathway for water flow may exist from medullary bundles to other regions in the plant body. However, experimental quantification of how much medullary bundles contribute to stem hydraulic conductivity remains unknown. This knowledge gap limits our ability to evaluate the ecological and evolutionary significance of medullary bundles.

In our present study, we investigated the development, structure, function, and hydraulic performance of medullary bundles in *Adelobotrys adscendens* (Melastomataceae), a climbing species from a tropical lowland rainforest (Schulman and Hyvönen, 2003). Medullary bundles have been reported in some member of Melastomataceae (Metcalfe and Chalk, 1950; Clausing and Renner, 2001a; Naranjo et al., 2018), making this species a potentially insightful example for understanding how such vascular variants emerge outside the lineages classically associated with arid or saline environments. By analyzing its vascular organization in relation to hydraulic conductivity, we aimed to clarify how medullary bundles are potentially integrated with the stem hydraulic system. We investigate the course of medullary bundles within the stem and the nodal vascular construction. We quantified the contribution of medullary bundles to stem hydraulic conductivity to assess their hydraulic contribution as compared to the secondary xylem. Our goal was to advance understanding of the hydraulic role of medullary bundles, and we later discuss how the functional integration of medullary bundle hydraulic performance and adventitious root development may have contributed to the evolution of root‐climbing and nomadic‐vine habits in humid environments.

## MATERIALS AND METHODS

### Growth form terminology

**Figure 1:**
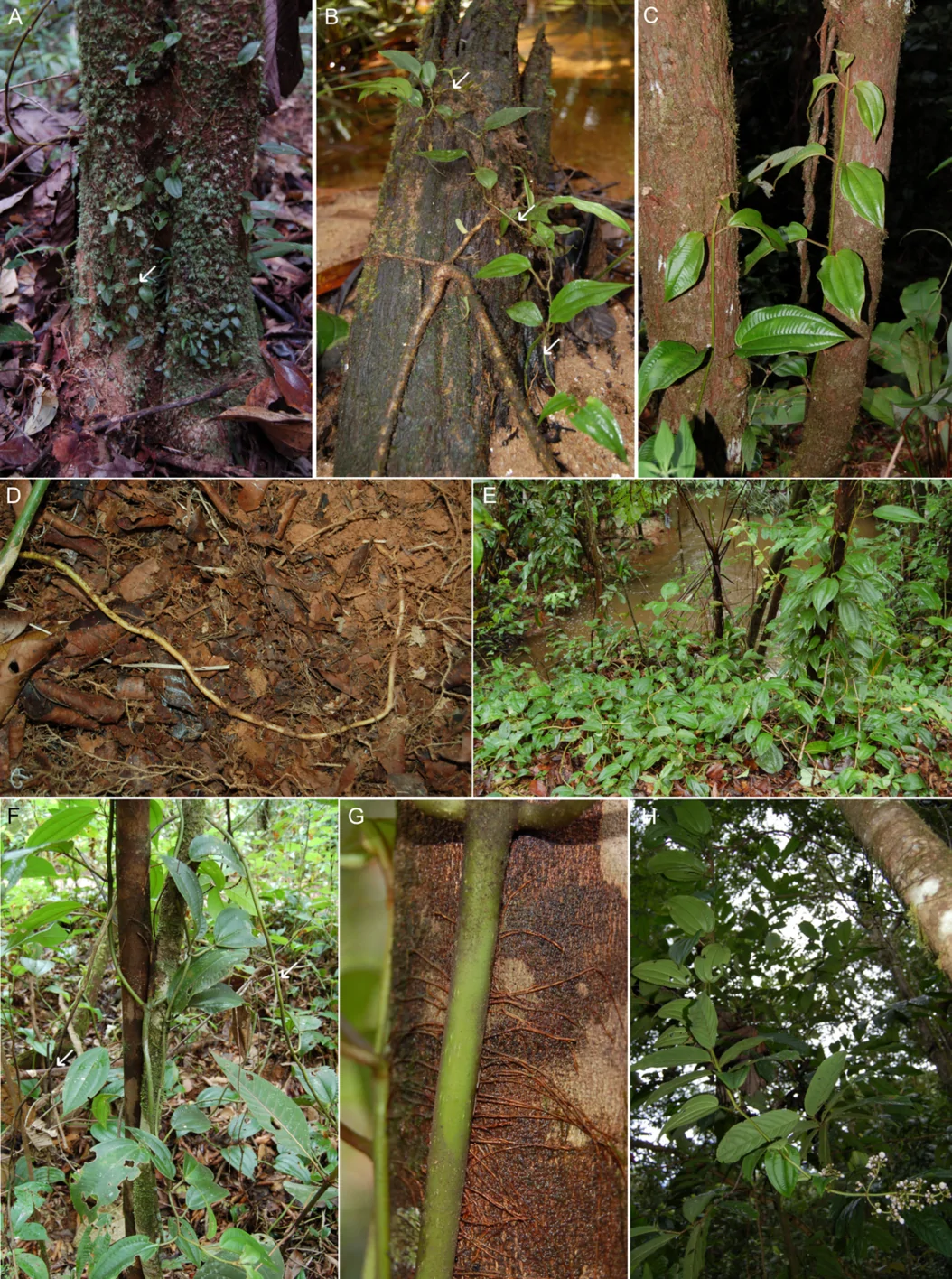
*Adelobotrys adscendens* in the lowland rain forest of French Guiana. (A) Seedlings climbing up from the base of a tree trunk. (B) A seedling growing on a log, and young stem hanging down toward the forest soil. (C) Climbing stems adhering to tree trunk via adhesive adventitious roots. (D) Old stem with long adventitious roots. (E) The species commonly forms a dense trellis of prostrate and climbing stems near rivers. (F) Stem of a nomadic vine with proximal senescence (left arrow) and hanging branches (right arrow) that will reach the forest soil. (G) Climbing stem adhering to a tree trunk via long adventitious roots. (H) Pendent branches with a terminal panicle in flower.

*Adelobotrys adscendens* (Sw.) Triana (Melastomataceae) has been variously described as a (1) “root climber” that climbs via adventitious roots to adhere to supports while a distal portion of the stem remains connected to the soil; (2) “hemiepiphyte”, a plant with seeds that germinate in the canopy to support and later establish root connection with the soil; or (3) “nomadic vine”, a climbing vine that has seeds that germinate on the ground and that loses the older part of the plant when ascending (Almeda, 1981; Clausing and Renner, 2001a; Zotz et al., 2021a). Our field observations indicate that the seeds germinate on or near the ground (on logs or trunk bases), the plants ascend the trunks and branches of other plants (8–10 m from the ground) by adhesive adventitious roots (Figure 1). Stems are always appressed to a support (often logs and the bases of large trunks), and no self‐supporting seedlings were observed (Figure 1C). Larger individuals branch intensively, and their branches hang and reach the soil where they re‐root (Figure 1F) or produce terminal panicle inflorescences that can dangle from the canopy (Figure 1H).

Hemiepiphytes (such as many aroids) establish connections with the ground later in their development (Zotz et al., 2021b). Such is not the case for *A. adscendens*. In *A. adscendens*, the stem maintains a continuous connection with the soil, and adventitious roots develop along the stem. As nomadic vines, the plant may shift position and lose the lower stem portion during growth (Moffett, 2000; Zotz et al., 2021b). We did not observe, however, that the plant climbs up and severs its connection to the ground. Thus, the life form of *A. adscendens* is best described as a root climber.

### Study site

Individuals of *Adelobotrys adscendens* were collected from the lowland tropical rainforest in French Guiana, in the vicinity of Piste de Saint‐Élie Research Station (5°17′ N, 53°03′ W; ~85 m a.s.l.). The study site is characterized by a lowland equatorial tropical climate and some seasonality of rainfall. Annual rainfall is ~3000 mm with a drier season from September to November (i.e., <100 mm per month). The monthly mean maximum temperature varies between 32.1–35.8°C, the monthly mean minimum between 19.5–20.8°C. Our work was done in March and April. Within the study site, three areas (each within 30 m of one another) were selected to sample a sufficient number of individuals (eight individual plants) for experimental work. Each site was located in the vicinity of small streams moving through mature forest.

### Hydraulic conductivity measurements

Branches were severed from plants using clippers, then placed into plastic bags with a small amount of water and transported to the laboratory (UMR Ecologie des Forêts de Guyane [EcoFoG], Kourou, French Guiana). In the laboratory, segments (3–12 mm in diameter) were cut from the branches (total of 36 stem segments from eight individuals) and stored in a wet bag until the hydraulic conductivity measurements.

Hydraulic conductivity of stems was measured as previously described (Sperry et al., 1988) (Appendix S1).

To tease apart the contributions of stem xylem versus the pith (medullary bundles) to hydraulic conductivity, we measured hydraulic conductivity of the stem (*K*_h stem_) and of the pith (*K*_h pith_; medullary bundles) alone (Appendix S1). After the measurement for each stem, the pith conductivity was measured as follows. (1) The outer cortex and wood were removed by cutting approximately 1 cm at both ends of the segment with a razor blade and gently scraping around the pith by hand. The pith, which is wide and vascularized, remains intact after the wood and cortex has been removed. (2) The trimmed segments were then wrapped with parafilm and attached to a soft plugging so that only the pith was connected to the hydraulic apparatus. (3) The stem was flushed as before but at 15 PSI for 20 min, then hydraulic conductivity was measured.

To determine which xylem conduits were conductive in the measured segments, we next perfused each segment with an aqueous solution of 0.05% w/v safranin. The safranin dye preferentially flows through conductive vessels and stains their cell walls.

### Theoretical hydraulic conductivity

A representative climbing individual was used to measure the frequency distribution of vessels in the stem xylem versus xylem vessels in medullary bundles over the developmental range of stem diameters sampled. Transverse cross sections (~40 µm thick) of the median portion of internodes were serially cut with a microtome with a vibrating blade (Microm HM 650 V; Thermo Scientific, Waltham, MA, USA) and imaged with an optical microscope (BX60; Olympus, Tokyo, Japan) interfaced with a digital camera (Olympus DP71). The contours of all vessels included in the medullary bundles and in the external vascular cylinder were drawn using Inkscape (https://Inkscape.org), and the area of each vessel was measured with Image J software (https://imagej.net; NIH Image, Bethesda, MD, USA). The circle equivalent radius of the area was used to calculate the theoretical conductivity of pith and external vascular cylinder for each segment, according to the Poiseuille law for ideal capillaries:

$$K_{\text{th}}=\pi \Sigma {r_{i}}^{4}/8\eta ,$$

where *K*_th_ is the theoretical conductivity (kg MPa^–1^ s^–1^ m), *r*_*i*_ is the radius of the *i*th vessel (m), *η* is the dynamic viscosity of the fluid (MPa s); the summation (Σ) is for all conducting elements. We then calculated the theoretical contribution of the pith to total theoretical hydraulic conductivity.

### Stem anatomy

Transverse cross sections (40 µm thick) were sectioned using a microtome with a razor blade (Microm HM 650 V) to measure the contribution of each tissue (pith, medullary bundles, external vascular cylinder) to the total area of the section. We also traced the trajectory of medullary bundles in the stem through a leaf trace by creating a video sequential images of 57 sections (each 40 µm thick) that from approximately a 5‐mm length of a node. Longitudinal sections of stems were cut by hand with a razor blade just after safranin dye injection to determine the paths of the medullary bundles across stem nodes.

Note that in the stele concept, the vascular cylinder includes the pith. Here, for simplification, we use the term “external vascular cylinder” to refer to the external part of the vascular cylinder, which is the only portion undergoing secondary growth. Thus, we distinguish the external vascular cylinder (that undergoes secondary growth) from the medullary bundles localized in the pith and that do not undergo secondary growth.

### Statistical analyses

Because multiple stem segments were sampled per individual plant, we fitted linear mixed‐effects models including individual as a random intercept to account for the nested structure of the data. Models were fitted using the R packages lme4 (Bates et al., 2015) and lmer (Kuznetsova et al., 2017) in R version 4.3.3 (R Core Team, 2024).

Allometric relationships between tissue area (pith and external vascular cylinder) and stem diameter were analyzed using log‐log linear mixed‐effects models.

Model fit for the mixed model was evaluated using the marginal and conditional *R* ^2^ to quantify the proportion of variance explained by the fixed effect and by both fixed and random effects, respectively.

## RESULTS

### Vascular architecture of medullary bundles

**Figure 2:**
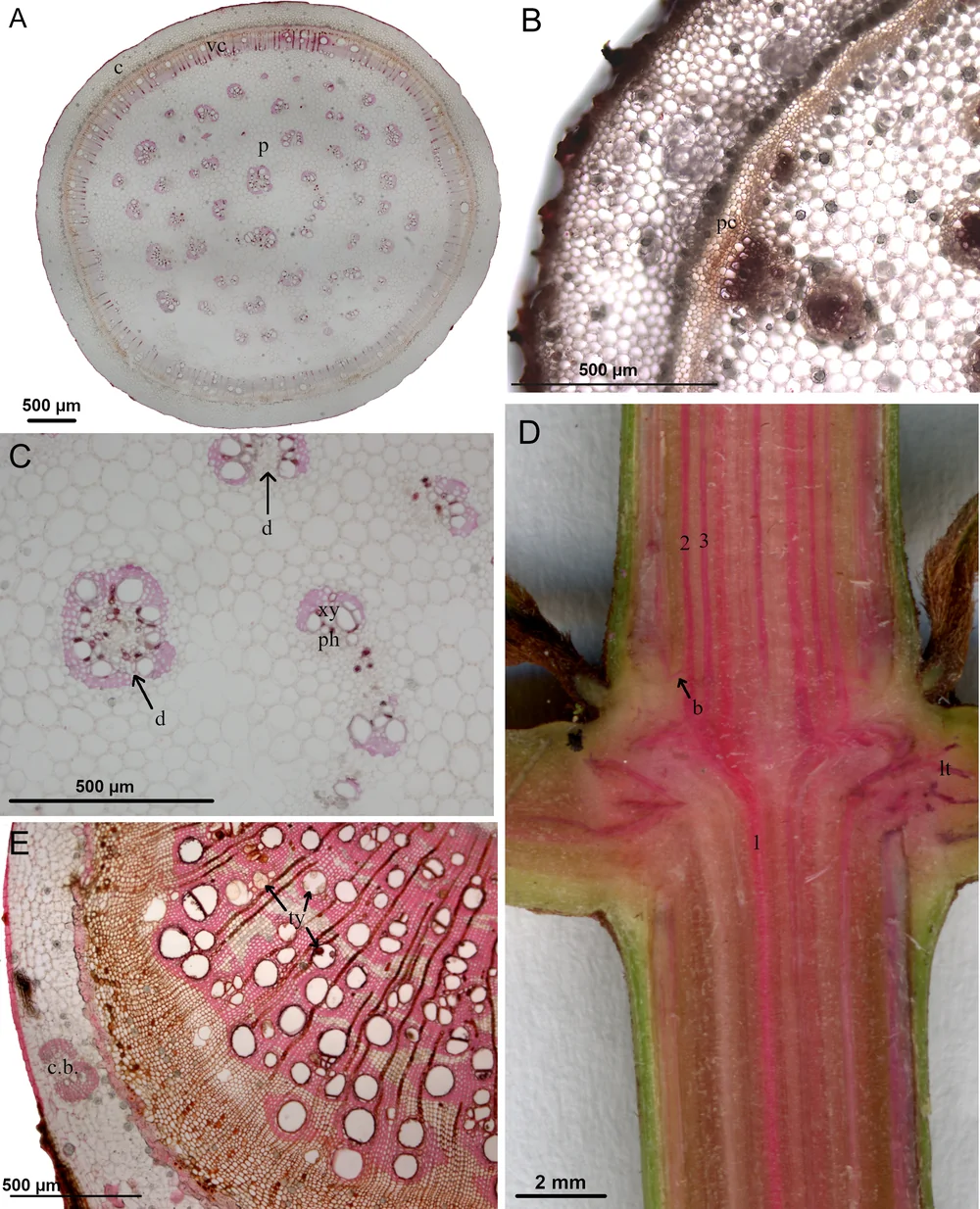
Stem anatomy of *Adelobotrys adscendens*. (A) Cross section of a stem, with active vessels stained with safranin. (B) Stem in early secondary growth with a medullary bundle and an external vascular bundle. The cambium is established from the vascular bundles formed by the continuous procambium. (C) Detail of cross section showing medullary bundles. Medullary bundles are collateral, having xylem and phloem on opposite sides. Some medullary bundles that appear amphivasal are actually medullary bundles undergoing division. (D) Longitudinal section stained with safranin, injected from the proximal end of the stem segment, showing nodal plexus. Peripheral medullary bundles curved outward as leaf traces; central medullary bundles have an axial trajectory (bundle 1) and bifurcate at node (bundles 2 and 3). (E) Old climbing stem with intense secondary growth. A cortical bundle is visible and results from medullary bundle entering the cortex during its course as leaf trace. b, bridge; c, cortex; c.b., cortical bundle; d, medullary bundle under division; lt, leaf trace; p, pith; pc, procambium; ty., tylose; vc, vascular cylinder; ph, phloem; xy, xylem.

In transverse section, the eustele of *Adelobotrys adscendens* is polycyclic and forms a continuous cambium that develops secondary tissues. Medullary bundles originate from procambial strands, and they are the first vascular tissues formed in the eustele. Overall, medullary bundles are scattered throughout the pith (Figure 2A). They are collateral, consisting of both xylem and phloem (Figure 2C). We did not observe any consistent orientation of the bundles relative to other stem tissues. Note that what appears to be amphivasal bundles are actually bundles in the process of division/anastomosis (see also the video in Appendix S2).

In longitudinal sections, medullary bundles anastomose and are bifurcate at stem nodes, forming a nodal plexus (Figure 2D). Central medullary bundles also follow an axial trajectory along the internode (Figure 2D) and bifurcate at the node, forming two bundles above the nodal region (Figure 2D). Bundle bifurcations are also observed in the transverse section (Figure 2C, arrows). The peripheral medullary bundles form leaf traces at nodes (Figure 2D). Short vascular connections, so‐called vascular bridges, which are comparable in structure to those described previously in monocotyledon stems (Zimmermann and Tomlinson, 1972; Vita et al., 2019), connect medullary bundles to the vascular cylinder (Figure 2D, arrow). In older stems, the vascular cambium produces relatively large amounts of secondary tissues, and bundles occur in the cortex (Figure 2E). These “cortical” bundles are leaf traces, which descend through the cortex for some distance before entering the pith. Tyloses were frequent in the secondary xylem, but were not observed in the medullary bundles (Figure 2E).

**Figure 3:**
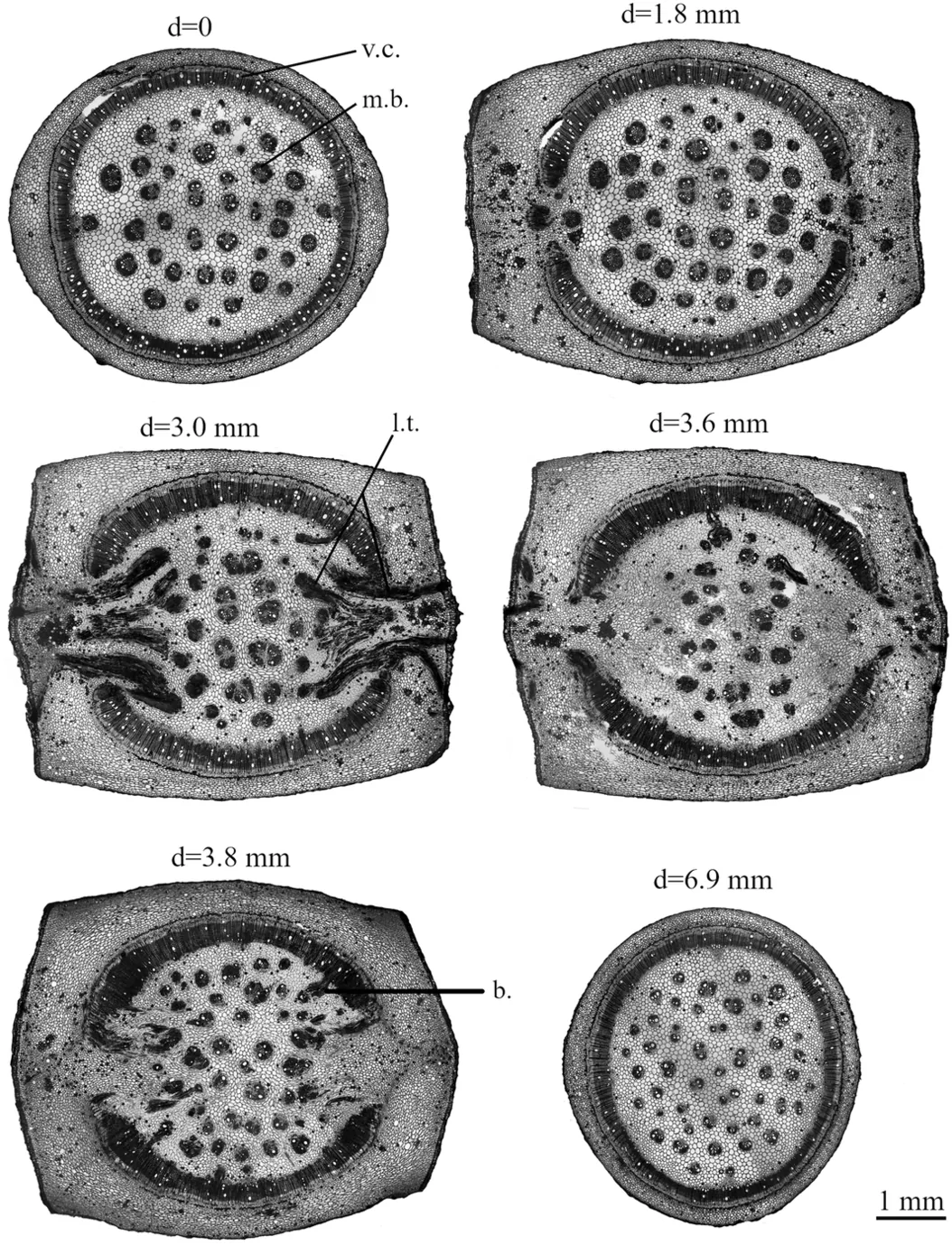
Series of cross sections (each 40 µm thick) upward along a node. The full sequence is available in Appendix S2. Medullary bundles enter a leaf gap as independent leaf traces. Distance (d) between each cross section is shown. v.c. vascular cylinder; m.b. medullary bundle; l.t. leaf trace; b. bridge.

**Figure 4:**
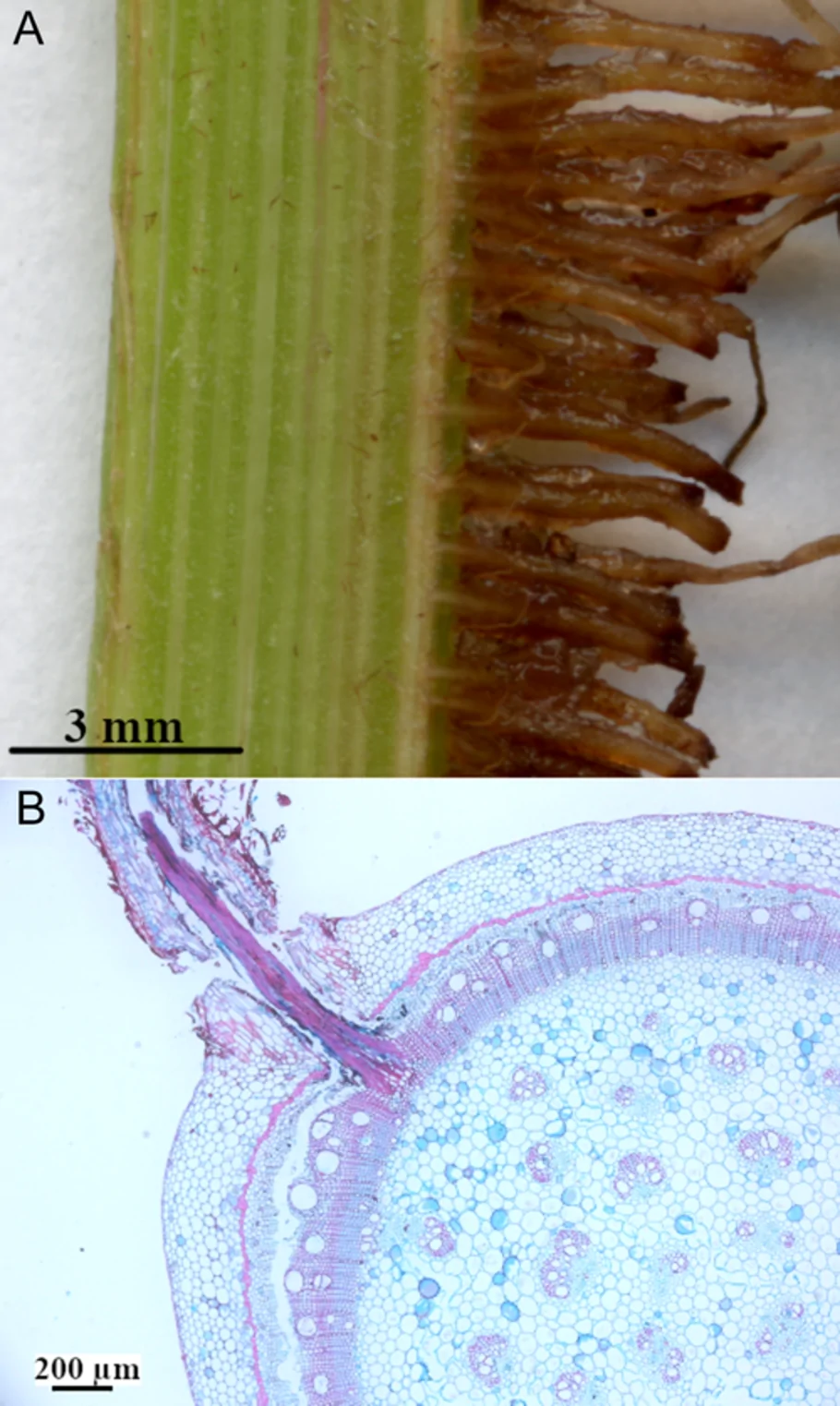
Stem sections showing anatomy of adventitious root of *Adelobotrys adscendens*. (A) Transverse section showing adventitious roots along an internode, with their connection to the vascular cylinder. (B) Cross section showing adventitious root extending from stem.

To trace the path of medullary bundles (Figure 3; Appendix S2), we cut a series of transverse sections over a nodal region. When approaching a node, the vascular cylinder becomes interrupted, forming a leaf gap. Peripheral medullary bundles diverge outward, forming leaf traces. Three to six leaf traces diverge on each side of the stem (opposite leaves). A few smaller peripheral bundles branch from the vascular cylinder (Figure 3). As a result of the departure of peripheral medullary bundles into the leaf traces, a deficit of bundles occurs approximately 500 µm above the last leaf trace. However, a relatively constant number of medullary bundles is maintained above the node through the bifurcation of the central medullary bundles at the node (Figures 2, 3). Thus, at the nodal level, the arrangement, size, and number of medullary bundles change as a result of bifurcation and departure as leaf traces. Abundant adventitious roots are produced along internodes (Figures 1, 4). The adventitious roots are continuous with the vascular cylinder (Figure 4B), but they are not structurally connected to medullary bundles.

### Hydraulic conductivity of medullary bundles

**Figure 5:**
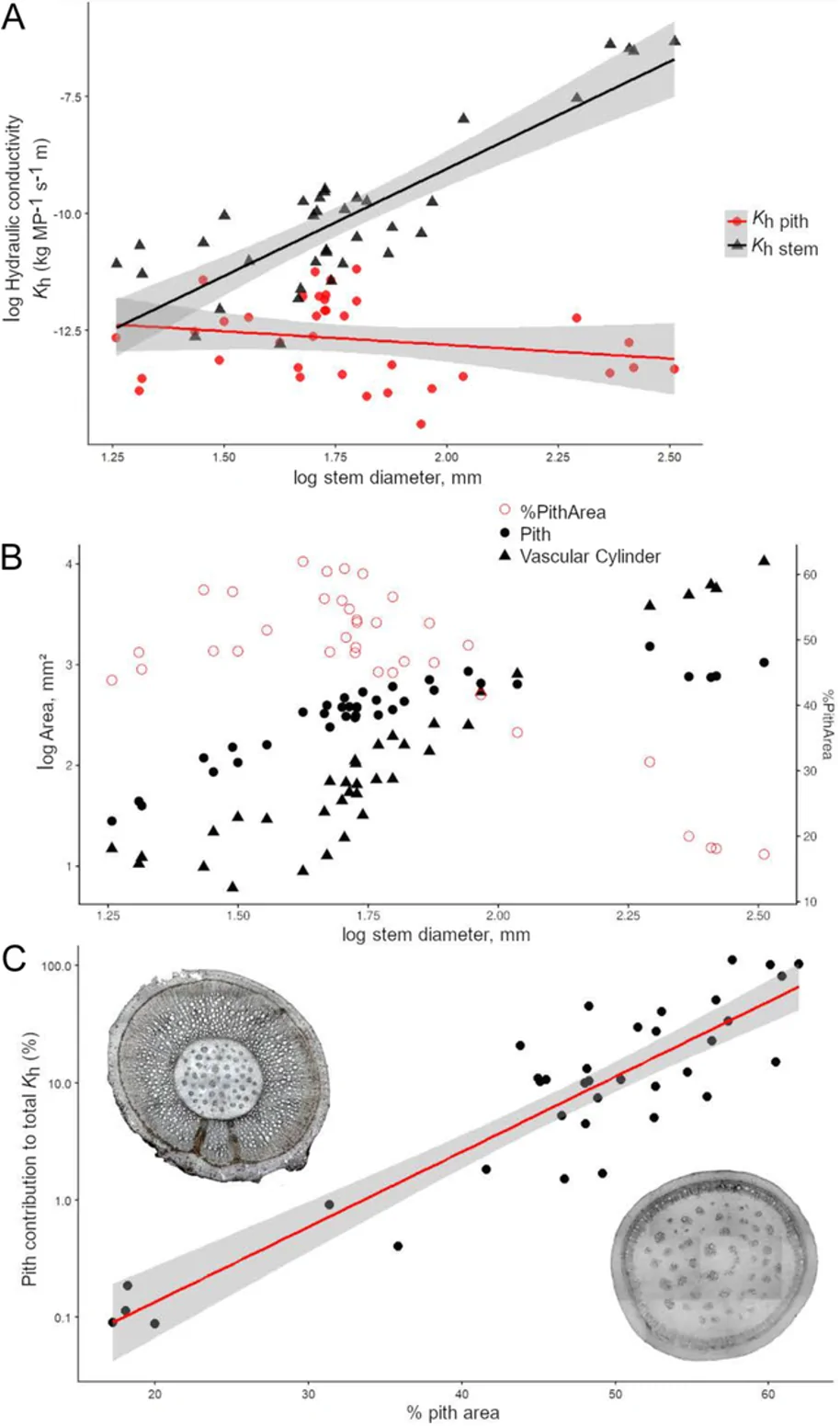
Hydraulic and geometric contribution of the pith. (A) Log‐log relationship between stem diameter and hydraulic conductivity, for the pith (*K*_h_ pith) and the stem (*K*_h_ stem). The red line shows the fitted linear regression, with 95% confidence interval (grey area). (B) Pith and wood area as a function of stem diameter (left *y*‐axis) and contribution of the pith to total stem area (right *y*‐axis). (C) Relationship between the percentage of pith area and the percentage contribution of the pith to total hydraulic conductivity. The red line shows the fitted linear regression, with 95% confidence interval (grey area). Note the *y*‐axis is on a logarithmic scale. Image at top left: cross section of a large stem. Image at bottom right: young apical stem with large pith and little wood development (*N* = 36).

Medullary bundles in stem segments that were 3–13 mm in diameter had a relatively limited but variable range of water transport capacities (10^–7^–10^–5^ kg s^–1^ MPa^–1^ m) with a mean conductivity of 4.4 × 10^–6^ kg s^–1^ MPa^–1^ m (SD = 3.6 × 10^–6^, *N* = 36). Stem hydraulic conductivity (*K*_h stem_) increased strongly with stem diameter, following a significant allometric relationship (*β* = 6.03 ± 0.52 SE, *P* < 0.001). In contrast, the hydraulic conductivity of the pith (*K*_h_ pith) showed no significant relationship with stem diameter (*β* = 0.51 ± 0.40 SE, *P* = 0.21) (Figure 5A), nor with pith area (pith area: *β* = 0.48 ± 0.40 SE; *P* = 0.237).

Pith area increased significantly with stem diameter following an allometric relationship, but less rapidly than proportionally (*β* = 0.71 ± 0.11 SE, *t* = 6.54, *P* < 0.001). The proportion of pith thus decreased in wider stems (diameters greater than ~6 mm; Figure 5B). In contrast, wood area increased strongly with stem diameter following a marked allometric relationship (*β* = 3.01 ± 0.16 SE, *t* = 18.27, *P* < 0.001), indicating a disproportionate increase of wood tissue in larger axes. The increases in stem hydraulic conductivity with stem size (Figure 5A) are primarily driven by the external vascular cylinder rather than by the pith.

The pith is large, making up ~60% of the stem cross section in young shoots (up to ~6 mm in diameter) to >15% in larger stems (~12 mm diameter) (Figure 5C).

The contribution of the pith to hydraulic conductivity increased significantly with the proportion of pith area. A linear mixed‐effects model revealed a highly significant positive relationship between percentage pith area and the pith contribution to total *K*_h_ (*β* = 0.127 ± 0.010 SE, *t* = 13.35, *P* < 0.001). The model explained a large fraction of the variance (marginal *R* ^2^ = 0.77; conditional *R* ^2^ = 0.89), indicating that pith fraction is a major factor of the pith contribution to the total *K*_h_. The contribution of the medullary bundles to the total stem conductivity was large and exceeded 80% in small‐diameter stems with a high fraction of pith area (>60%) (Figure 5C). In larger‐diameter stems, where pith contribution to the stem surface was the lowest, medullary bundles contributed very little to axial hydraulic flow (<1%).

**Figure 6:**
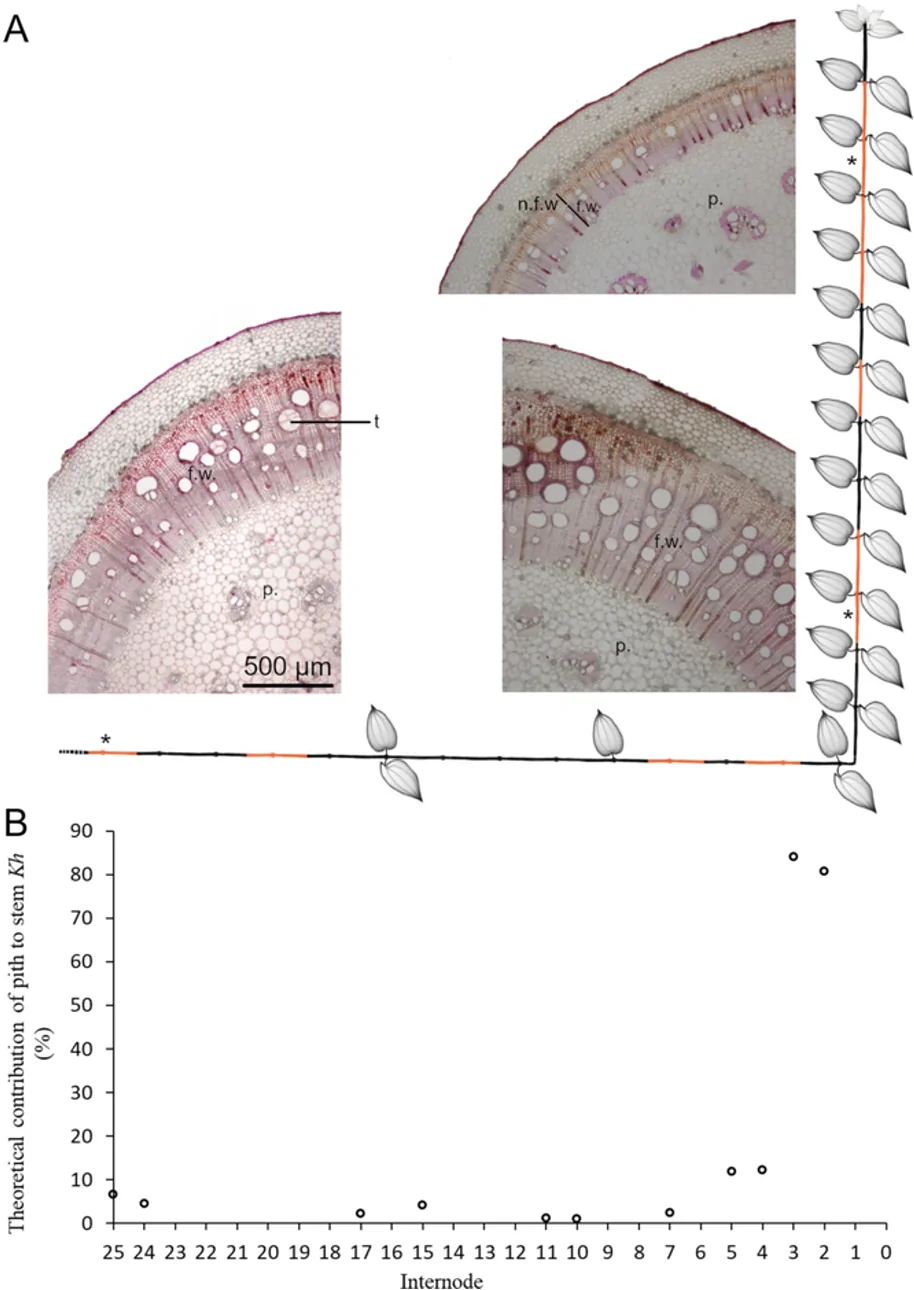
Theoretical contribution of the pith to stem conductivity. (A) Diagram of representative climbing plant, with a prostrate and climbing portion of stem. Orange segments indicate the position of the internode segment measured and shown in panel B. Asterisks indicate the position of three stem transverse sections, cut after the safranin injection and showing the conductive part of the stem. (B) Theoretical contribution of pith to the total stem conductivity along stem internodes, from tip to base. f.w., functional wood; n.f.w., nonfunctional wood; p., pith; t., tylose.

A representative climbing plant (Figure 6) with steady diameters (5.13 mm ± 0.35) was used to calculate variations in *K*_h_ along the stem, from the plant apex. The first internode was excluded due to its minute size and incomplete development. When the theoretical contribution of medullary bundles to total *K*_h_ was plotted along the internode path, values for the second and third internode equaled more than 80% of the total *K*_h_ (Figure 6B). This contribution dropped drastically in the fourth and fifth internodes to about 10% and was insignificant in the next internodes, where wood takes over conductivity. In the first internode measured, young vessels of secondary wood were not functional (safranin staining, Figure 6A).

**Figure 7:**
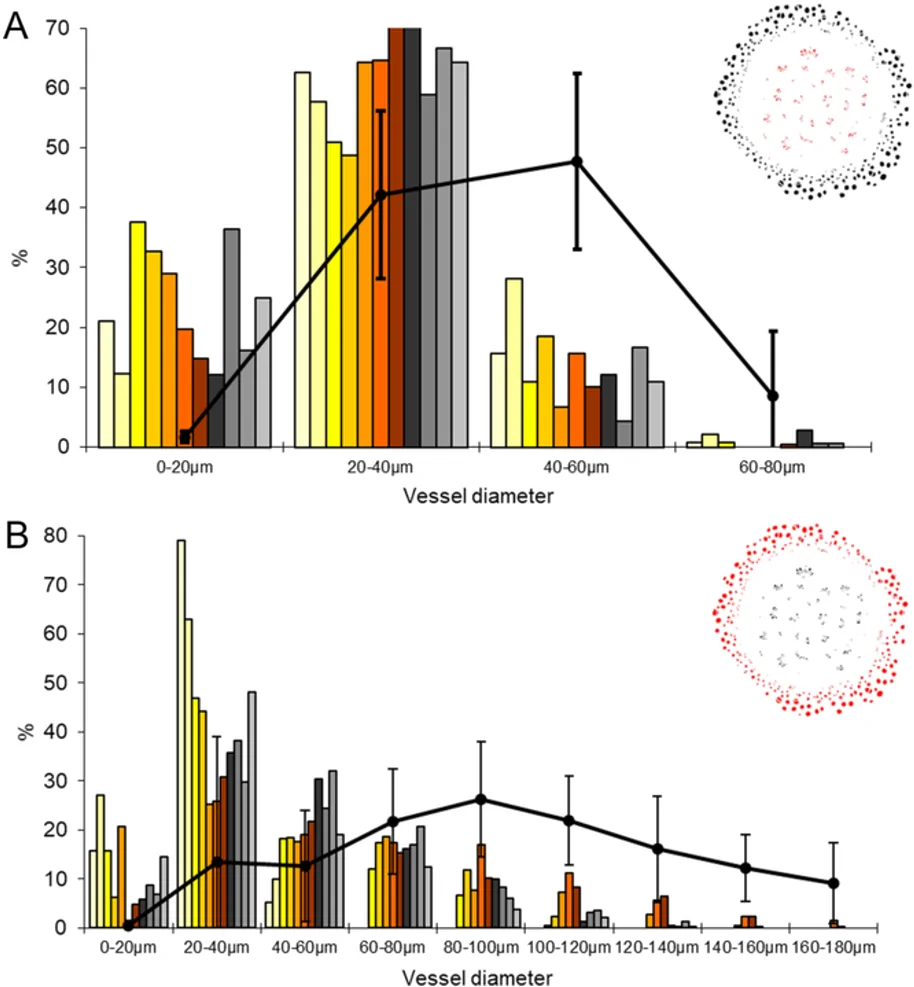
Frequency distribution (%) of vessel diameter (bars) and theoretical contribution (%) to total conductivity *K*_h th_ (mean ± SD, *N* = 11) for each vessel diameter class, for (A) medullary bundle and (B) wood in a stem of *Adelobotrys adscendens*. The *y*‐axis therefore represents the percentage of each diameter class in a stem cross section (bar) and the contribution of this diameter class to the stem theoretical conductivity (black line). In the drawings, the measured vessels are colored in red.

**Figure 8:**
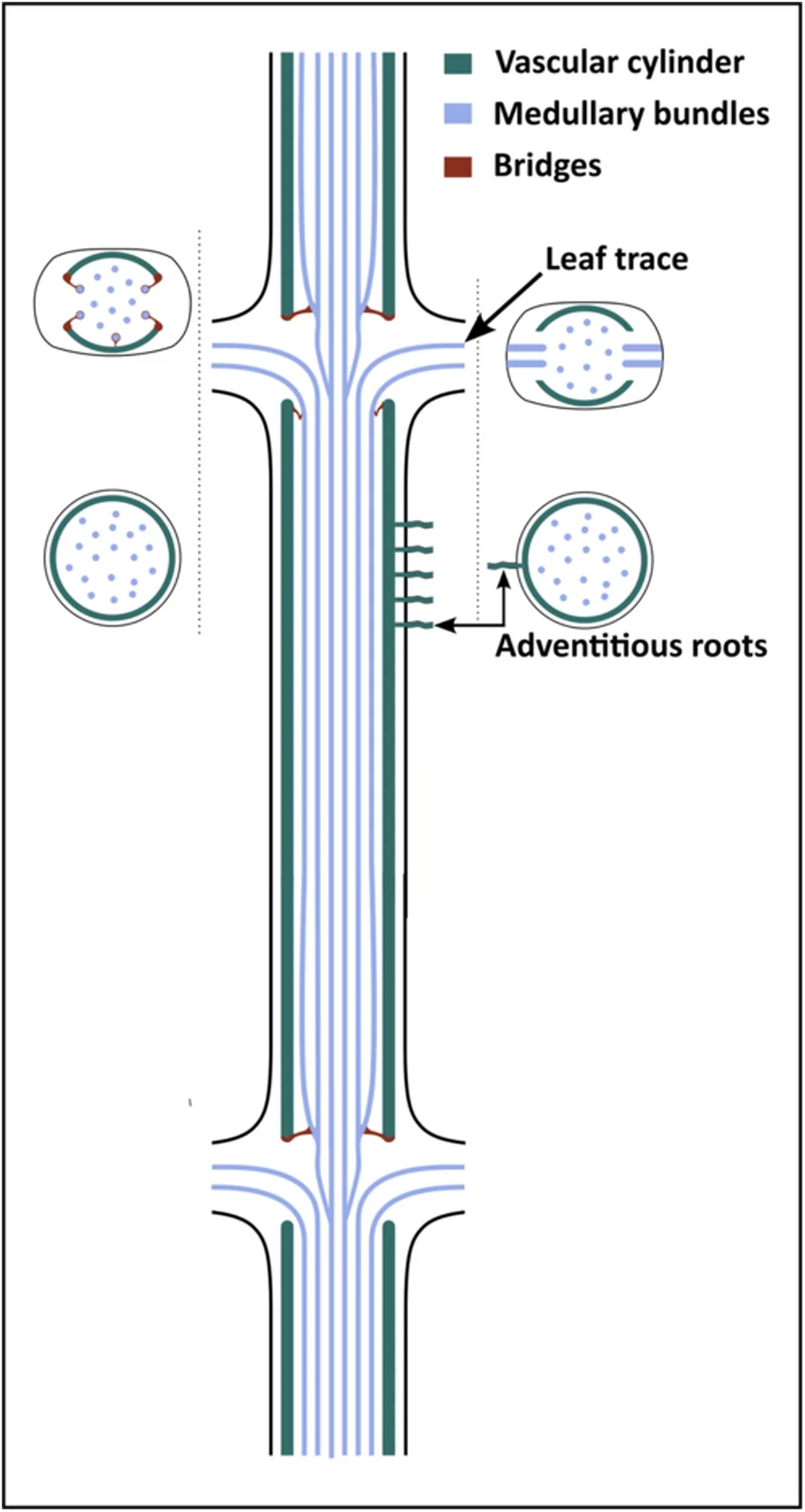
Schematic representation of the course of medullary bundles in the phytomer. Axial medullary bundles, in the center of the pith, bifurcate at nodes, and peripheral bundles give rise to leaf traces. Bridges link the vascular cylinder to medullary bundles at nodes. Adventitious roots, developing along internodes, are connected to the vascular cylinder.

In medullary bundles (Figure 7A), about 60% of vesselswere within the diameter class 20–40 µm. Larger diameter vessels (40–60 µm) were less frequent but explained almost 50% of the theoretical *K*_h_. Vessel diameters never exceeded 80 µm in medullary bundles, explaining the limited range of hydraulic conductivity of this hydraulic system. For wood, vessel size frequency was positively skewed, with many narrow vessels (20–40 µm) and pronounced tails to wide diameter vessels up to 160–180 µm. Vessels with a diameter greater than 80 µm (only ~13% of vessel frequency) contributed to more than 60% of the theoretical *K*_*h*_ (when the contribution of each diameter class above 80 µm was totaled) (Figure 8).

## DISCUSSION

### Contribution of medullary bundles to axial water transport

Our hydraulic conductivity experiments demonstrated an important function of medullary bundles in hydraulic conductivity in the stem. We found that the medullary vascular system represents a critical component of axial water transport in the plant apex before the external vascular cylinder becomes fully functional. In *A. adscendens*, this contribution is most pronounced in young climbing stems (<6 mm in diameter) and in the apical portion of shoots (first to third/fourth internodes), where medullary bundles account for up to 80% of both measured and theoretical maximum stem conductivity. A recent study demonstrated that medullary bundles can theoretically conduct water (Cunha Neto et al., 2024). However, our work provides, to our knowledge, the first direct experimental quantification of water flow capacities through medullary bundles.

Compared with Cunha Neto et al. (2024), who reported a potential (theoretical) contribution of up to 96.2% (based on theoretical calculations, not direct measurements), near the apex in Caryophyllales, our estimates are slightly lower. The difference may reflect differences between herbaceous species, where medullary bundles maintain a higher contribution, versus a species developing secondary growth, where their role decreases markedly with stem development. In the study by Cunha Neto et al. (2024), two of the three species examined were herbaceous and retained a theoretical contribution close to 50%, whereas this contribution dropped to nearly zero in *A. adscendens*. In *A. adscendens*, the relatively low value of medullary conductivity (4.4 × 10^−6^ ± 3.6 × 10^−6^ kg s^−1^ MPa^−1^ m; *N* = 36) results from the narrower vessel diameters (<80 µm) in medullary bundles as compared to the much wider vessels distributed in the external vascular cylinder (up to 180 µm diameter).

### Vascular construction and hydraulic segmentation

Medullary bundles of *A. adscendens* originate from procambial strands formed during primary growth near the shoot apex (Type I sensu Cunha Neto et al., 2024). They are regularly arranged and in two main forms: (1) peripheral bundles that give rise to leaf traces and (2) axial bundles that cross and bifurcate at the nodes. The departure of medullary bundles as leaf traces in Melastomataceae was noted by Metcalfe and Chalk (1950), who reported that many medullary bundles enter the petiole independently of the external vascular cylinder. Our observations extend this observation by demonstrating that, in *A. adscendens*, medullary bundles form the sole vascular supply entering the petiole. Thus, medullary bundles represent an alternative conductive feature than the typical leaf traces formed by the vascular cylinder in most woody angiosperms (Esau, 1958). However, medullary bundles do not constitute an independent vascular system within the stem because they are connected to the peripheral vascular cylinder at the node via anastomoses and vascular bridges, comparable to those described in monocotyledon stems (Zimmermann and Tomlinson, 1972; Vita et al., 2019). Vascular bridges have also been described in Nyctaginaceae, many of which are from arid environments (Cunha Neto et al., 2020, 2022).

The vascular cylinder is also connected to adventitious roots that develop along internodes, as commonly observed in woody angiosperm species, in which the adventitious root vasculature is integrated with that of the stem (Groot et al., 2003). We hypothesize that, in some circumstances, local uptake and transport of water and nutrients through adventitious roots may be more important than long‐distance axial transport from root systems in the ground. Although we could not test this idea because the diminutive size (<1 mm diameter) of these roots prevented hydraulic conductivity measurements, their functional contribution is plausible. The main function of adventitious roots in root climbers is mechanical adherence to the support (Steinbrecher et al., 2010), but does not preclude a conductivity function. For example, aroid vines produce distinct root types: anchor roots and feeder roots (López‐Portillo et al., 2000; Rasmussen et al., 2019). Anchor roots in the epiphytic *Rhodospatha oblongata* were shown to be hydraulically functional, albeit with low conductivity (0.01–2.7 × 10^−6^ kg s^−1^ MPa^−1^ m) (Filartiga et al., 2017), comparable to the values we obtained here for medullary bundles. Likewise, nutrient uptake by aerial adventitious roots has been demonstrated, and nutrients absorbed by these roots remain localized close to the point of entry (Nadkarni and Primack, 1989). Adventitious roots thus provide localized access to water and nutrients that can sustain the medullary bundles independently from the ground‐root system.

### Hydraulically segmented phytomers: a modular view of vascular function

The vascular organization observed in *A. adscendens* suggests that stems may operate as a series of hydraulically segmented phytomers, each integrating leaves, node, internode, and adventitious roots into a semiautonomous hydraulic unit. This modular organization echoes the concept of hydraulic segmentation (Zimmermann, 1978; Tyree and Ewers, 1991), but at a finer scale, where each phytomer forms a localized hydraulic domain. In this framework, water and nutrients are supplied locally through adventitious roots, reducing reliance on long‐distance axial transport from ground roots. The short transport distance within the phytomer and the predictable hydraulic constriction at the stem–leaf junction (Holbrook and Sack, 2006; Levionnois et al., 2020) mean that the relatively low conductivity of the medullary bundles is not a constraint to overall hydraulic functioning at this small scale. Rather, their integration with the nodal vascular network ensures localized water transport and redundancy, maintaining water flow even when proximal segments are hydraulically disconnected, for example, after stem proximal decay. Such a design enhances hydraulic resilience and modularity, traits likely advantageous for species with root‐climbing or nomadic vine habit, where ground‐rooted connections are subject to frequent disturbance during ontogeny (Zotz et al., 2021a, b). This hydraulic construction could also promote vegetative reproduction through fragmentation. In *A. adscendens*, phytomers may define discrete hydraulic units, thereby reducing reliance on long‐distance stem transport and promoting resilience during ontogenetic transitions. The hydraulic chokepoints and low conductivity imposed by the sudden shift in proto‐/metaxylem transport in medullary bundles is probably not a physiological constraint in wet habitats involving generally low evaporative demand.

### Medullary bundles might increase stem capacitance

Beyond water and nutrients transport, medullary bundles have also been hypothesized to contribute to storage functions, as in Cactaceae (Mauseth, 1993). The broad pith and associated parenchyma may act as a hydraulic capacitor, particularly during the vulnerable shift from prostrate to tree‐ascending growth. *Adelobotrys adscendens* lacks heartwood, and the broad pith persists during stem development. These observations suggest that medullary bundles may serve to store water and possibly other resources such as starch. We observed numerous parenchyma cells surrounding medullary bundles containing starch grains. Similar observations were made by Cunha Neto et al. (2020) in plants from dry environments.

### Vascular architecture and life form

Our field observations indicate that seeds of *A. adscendens* germinate on or near the ground (on fallen trunk), and individuals lose the proximal portion of their stem during growth. However, the stem maintains soil contact through adventitious roots that develop along the nodes at ground level. This type of development means that *A. adscendens* grows at the intersection of nomadic vines (Moffett, 2000; Zotz et al., 2021b) and root climbers (Almeda, 1981). A strict root climber would keep the primary root system, and the difference is important because nomadic vines shift position and lose the lower stem portion. The distinction between root‐climbing and nomadic vines is not trivial. Clear terminology is crucial for describing community structure and understanding evolutionary transitions among climbing epiphytes (Zotz et al., 2021a; Hoeber and Zotz, 2022; Einzmann et al., 2024). In our study, understanding the life form of *A. adscendens* is key to evaluating the selective advantage of medullary bundles.

Almost all so‐called nomadic vines that have been reported are monocotyledons (Zotz et al., 2021a). More broadly, monocots, with their scattered bundles and prolific adventitious rooting, dominate epiphytic niches worldwide (Gentry and Dodson, 1987; Benzing, 1990; Carlquist, 2012). Nomadic vines lose their primary connection with the soil (they lose the proximal portion of the stem), then rely on a secondary connection (adventitious roots), that bypasses long‐distance stem transport. In monocotyledons, this hydraulic strategy is an adaptive response to the lack of secondary growth; the development of adventitious roots is the only option to meet the increase in hydraulic demand as the shoot increases in size (López‐Portillo et al., 2000). Thus, a common hydraulic trait of nomadic vines is that water flows directly from the ground to the internode/node, through adventitious roots. In addition, large pith in monocots provides greater water storage (capacitance).

In epiphytic monocots such as orchids and bromeliads, the scattered vascular bundles are combined with water‐storage tissues and prolific adventitious roots, hypothesized to provide hydraulic resilience against episodic droughts that occur frequently in canopy habitats (Benzing, 1990; Meyer and Zotz, 2004; Riordan et al., 2023). Araceae (hemiepiphytic and nomadic vines members) species possess rather succulent stems, suggesting high capacitance. During periods of water stress, such as during the dry season or when feeder roots are damaged, the plant may depend on water stored in its stem for survival.

The relationship between life form and vascular construction also occurs in other lineages besides monocots. In *Peperomia* (Piperaceae, Magnoliidae), for instance, where epiphytic habits have evolved numerous times (Samain et al., 2009), secondary growth is strongly reduced or absent, and the loss of peripheral bundles may have resulted in a monocot‐like organization with scattered medullary strands (Isnard et al., 2012). In other non‐monocot woody angiosperms, convergent solutions, such as the development of medullary bundles linked to aerial rooting, may also facilitate the evolution toward epiphytic or hemiepiphytic habit (e.g., Piperaceae). From this perspective, the monocot‐like hydraulic organization of *A. adscendens* can be interpreted as part of a broader functional trait complex, where medullary bundles and adventitious roots form “hydraulically segmented phytomers”, jointly facilitating the transition to epiphytic/nomadic vines.

Within Melastomataceae, medullary bundles occur in several epiphytic or root‐climbing lineages (Clausing and Renner, 2001a), but a systematic assessment is needed to test the potential correlated evolution of medullary bundles and the epiphytic/root‐climbing habit. The family is a relevant model group to test evolutionary hypotheses because a wide range of growth forms and a significant number of the world's epiphytes and climbers are represented in the family (Renner, 1986; Clausing and Renner, 2001b; Zotz et al., 2021a).

Xylem architectural modularity may have allowed some root climbers and nomadic vines to evolve with less investment in secondary growth and a long axial pathway. Reducing hydraulic and carbon costs is crucial for nomadic vine taxa because they are skototropic (Strong and Ray, 1975), meaning they grow toward shade rather than light. Thus, reducing carbon costs is adaptive because carbon availability for growth is limited in the understorey.

This anatomical–functional association deserves closer attention because it may represent a recurrent evolutionary pathway among non‐monocot angiosperms adapting to canopy life. Future studies on water relations and vascular architecture in hemiepiphytes and nomadic vines should explicitly address the contribution of medullary bundles in shaping these ecological transitions and evolutionary trajectories.

### Diversity of medullary bundles and their functional implications

Although medullary bundles have been reported in nearly 40 families, the term encompasses a heterogeneous set of structures that do not necessarily share the same developmental or evolutionary origins (Wilson, 1924; Mauseth, 1993; Evert, 2006; Quijano‐Abril et al., 2013). In Caryophyllales, for instance, three categories of conducting tissues within the pith have been described—procambial, extrafascicular, and perimedullary strands (Cunha Neto et al., 2024). Among them, only those of procambial origin correspond to “true” medullary bundles, representing more than 80% of the conducting vascular tissues in the pith (CVTP) reported in the order (Cunha Neto et al., 2024: Table 1). Most occurrences are concentrated in herbaceous species, (root) climbers, or epiphytes, consistent with a developmental link between unusual vascular organization and life forms.

Other types of vascular elements in the pith may originate asynchronously, often after secondary growth has already been initiated (Cunha Neto et al., 2024). These units, sometimes referred to as “conducting vascular units”, may participate in storage (water or starch) rather than in hydraulic transport, suggesting that not all medullary tissues are equivalent, from a hydraulic perspective.

This structural diversity suggests that medullary bundles do not represent a single anatomical entity but a spectrum of vascular arrangements with potentially distinct ecological roles. Understanding the developmental origins of these bundles is therefore crucial for interpreting their possible adaptive significance. Fossil evidence indicates that pith vascularization is an ancient trait, with medullary‐like vascular tissues reported in several fossils (Xu et al., 2017; Decombeix et al., 2019; Cunha Neto et al., 2024). Notably, cladoxylopsid trunks combined medullary vascular networks with abundant adventitious roots (Xu et al., 2017), echoing the recurrent association observed in several extant climbers and epiphytes. Such findings underscore that the medullary bundles may have played recurrent roles in plant hydraulics through deep time. Their inclusion is therefore essential when assessing xylem evolution in living and in fossil plants.

## CONCLUSIONS

Our study highlights the overlooked yet significant role of medullary bundles in the hydraulic architecture of *Adelobotrys adscendens*, contributing up to 80% of axial hydraulic conductivity in young axes and apical shoots before the onset of secondary growth. As secondary xylem develops at the periphery of the stem, pith contribution to axial water transport rapidly declines, indicating a primarily transient but functionally critical role during early stem development. Beyond contributing substantially to axial water transport in young axes and apical shoots, medullary bundles also connect directly to leaves, influence stem–petiole hydraulics, and may provide complementary functions such as increasing stem capacitance, thus buffering embolism. Their organization resembles the vascular systems of monocots and appears closely tied to the root‐climbing and partially nomadic vine habit of the species. We suggest that medullary bundles, together with adventitious rooting strategies, form part of a functional trait complex facilitating hydraulic resilience and possibly predisposing some eudicots and magnoliids to epiphytic or nomadic‐vines niches. The concept of hydraulic units, functioning at the phytomer level and implying local water uptake, needs further investigation. Further comparative studies are needed to clarify their evolutionary significance across lineages.

## AUTHOR CONTRIBUTIONS

S.I.: design of the research; S.I., D.L.: data collection; D.L., S.I.: anatomical data; S.I., D.L., T.F.: data analysis and interpretation; S.I., T.F.: writing original draft; S.I., D.L., T.F.: review and editing.

## Supporting information


**Appendix S1:** Conductivity apparatus and methods for measuring the hydraulic conductivity and contribution of the pith.

**Appendix S2:** Video illustrating the trajectory of medullary bundles across a node. The video was created using sequential image stacking of 57 sections (total thickness: 40 µm) from approximately a 5‐mm length of a node.

## Data Availability

Data are available at Zenodo: https://zenodo.org/records/22231226.
