# Supplementary material for: Hydraulic function of medullary bundles in a tropical root climber
Source: Am J Bot. 2026 Sep 4;113(9):e70256. doi: 10.1002/ajb2.70256 (PMC13595498; doi:10.1002/ajb2.70256)
Supplement: Supplementary file 1 [file AJB2-113-e70256-s001.docx]

**Appendix S1.** Conductivity apparatus and methods for measuring the hydraulic conductivity and contribution of the pith.

We built a conductivity apparatus using the methods of Sperry et al. (1988). Stem segments were cut underwater, wrapped with Teflon film, and attached to soft silicone tubing filled with water before attaching to the conductivity apparatus. A fresh razor blade was used to excise the stem end underwater before attaching a stem segment to the perfusion hydraulic apparatus. To tease apart the hydraulic contributions of stem xylem versus that the hydraulic conduits of the pith (medullary bundles), we first measured hydraulic conductivity of the stem (*K*_h stem_) and then of the pith alone (*K*_h pith_).

To measure the conductivity of the pith, we removed the outer cortex and wood, approximately 1 cm from each end of the measured segment by first slitting through the cortex and wood using a razor blade and gently removing the outer tissues, leaving only the pith. The pith, which is wide and vascularized, remains intact and whole after the removal of the outer tissues (wood and cortex). The segments were wrapped in parafilm and attached to silicone tubing so that only the pith was connected to the hydraulic apparatus.

For the measurements, stem segments were first flushed for 1 h at 150 kPa with a degassed 0.01 M KCl aqueous solution, which had been passed through a 0.2-mm filter, to remove possible xylem emboli. The maximal hydraulic conductivity (*K*_h_) was then determined using a lower pressure (~3.5 kPa) with water flow (*F*, kg s^–1^) through the segment measured with an analytical balance (±0.1 mg) (Mettler AE200, Mettler Toledo, Columbus, OH, USA). *K*_h_ (kg s^–1^ MPa^–1^ m) was calculated as *FL*/d*P* where *L* = the length of the segment (m) and d*P* = the pressure gradient over the measured stem.
